# Anthropometric, Physical, and Age Differences by the Player Position and the Performance Level in Volleyball

**DOI:** 10.2478/hukin-2014-0128

**Published:** 2014-12-30

**Authors:** José M. Palao, Policarpo Manzanares, David Valadés

**Affiliations:** 1 Department of Physical Activity and Sport, Faculty of Sport Science at the University of Murcia, Spain.; 2 Athletic Department, Catholic University San Antonio, Murcia, Spain.; 3 Department of biomedical sciences, Faculty of Medicine and Health Sciences at Alcalá University, Alcalá de Henares. Madrid, Spain.

**Keywords:** performance, evaluation, team sport, role

## Abstract

The purpose of this paper was to study the ranges in height, weight, age, spike reach, and block reach of volleyball players in relation to the player position and the level of their respective teams in peak performance. The analysed sample included 1454 male and 1452 female players who participated in the volleyball competitions of the Olympic Games and World Championships in the 2000–2012 period. A descriptive, correlational, and longitudinal design was used. The variables studied were: the player position, body height, weight, body mass index, spike reach, block reach, age, and team level. The results show differences between body height, spike and block reaches, and the age of the players by their position. These differences are related to the needs of the different positions with regard to the actions they execute. Middle-blockers, outside-hitters, and opposites have the characteristics that are most suitable for blocking and spiking, and the setters and liberos appear to have characteristics conducive to setting and receiving as well as digging, respectively. The differences found in the studied variables with regard to the playing position are related to players’ needs regarding the actions they perform. Player’s age was a variable that differentiated first teams at this level of competition for males, and physical capacities (body height, weight, spike reach, and block reach) were variables that differentiated first teams at this level of competition for females.

## Introduction

Performance in sport depends on a combination of technical, tactical, physical, psychological, and anthropometric factors (Bompa, 1999; Grosser and Neumaier, 1986). In volleyball, the anthropometric characteristics of the players are an important aspect in peak performance due to existence of an obstacle that players must overcome: a net that is 2.43 m high for males and 2.24 m high for females. The anthropometric characteristics and technical abilities determine 83% of the reach in a player’s jump, and the physical capacities determine 17% (Vint, 1994). The jump reach is fundamental in performing the spike and block. In volleyball, importance of the anthropometric, physical, and technical aspects increases because the spike, both for the men’s and women’s game, and the block, in men’s game, are the actions that are most correlated with winning (Eom and Schutz, 1992; Palao et al., 2004).

Player participation in the spike and block changes in relation to the player’s role, because players have different actions and responsibilities with regard to their position (Table 1). For example, the middle blockers are the players that execute the most blocks, so, in theory, they should have adequate anthropometric and/or physical characteristics to fulfil this role. On the other hand, the setter and the libero do not need to be as tall or strong (Fattahi et al., 2012), but they require more experience for correctly reading the game and for decision making and more agility.

**Figure 1:**
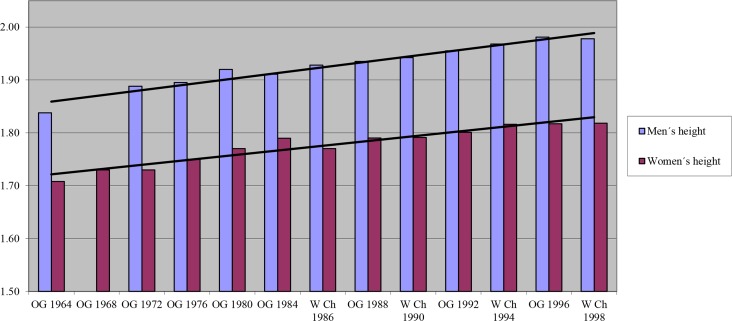
Evolution of player’s body height in the Olympic Games and World Championships from 1964 to 1998 (data given in meters and obtained from: Alberda, 1995; Baacke, 1989; Ejem, 1991; Fröhner, Zimmerman and Kügler, 1997; Gérard et al., 1991; MacLaren, 1990; Mountinho; 2000; Murphy, 1995; Meiner and Sawula, 1991; Sawula, 1991).

In the last few decades, there has been an increase in body height of the players that participated in the World Championships and in the Olympic Games (Figure 1). However, the majority of the previous studies have not taken into consideration the player’s position or player’s competition level.

Gualdi-Russo and Zaccagni (2001) studied somatometric components of elite male (n=234) and female (n=244) volleyball players in relation to their different game positions and levels of competition in the Italian League (first and second divisions). In the 1992–1993 and 1993–1994 seasons, they found differences by a level of competition (first division vs. second division) and by the player’s position for both genders. At a higher level of competition, higher ectomorphic traits and lower endomorphic and mesomorphic traits were found. In first division male players, there was a slight tendency toward greater homogeneity in somatotype. By a playing position (Table 2), differences were found between the setter and the rest of the positions (centres, attackers, and opposites) and between middle-blockers and the rest of the players (setters, attackers, and opposites). For both genders, setters presented highest mesomorphic values and centres presented highest ectomorphic values. Similar tendencies were found for international male players (n=35) (Marques et al., 2009). They found that middle blockers and opposite players were taller, heavier, and stronger than setters and liberos. Similar results were found for female Greek volleyball players (n=163) by Malousaris et al. (2008). They also found that players of higher levels (A1 vs A2) were taller and had a lower BMI. The same tendency was found for female volleyball players of the first Spanish Division (n=147), in relation to final team classification (Martín-Matillas et al., 2014). Another study by Sheppard et al. (2009) found that male middle blockers were taller and heavier than outsides and setters (n=142). Similar tendencies were found by Carbajal et al. (2012) in a study about the Cuban women’s team (n=41) in the Olympic Games of 1992, 1996, and 2000. In these last two papers, the libero position was not taken into consideration.

In 1998, the Fédération Internationale de Volleyball (FIVB) introduced new rules and created a new player position, the libero. This player is a specialist in reception and defence. The libero is restricted to playing in the back court and cannot serve, set inside of the 3 meter line, or spike. The FIVB introduced this figure in international competitions with the objective of allowing shorter players to play in peak performance. This is demonstrated by the 2002 Women’s World Championship when Italy won the final with a libero measuring 1.62 m while the average height of the rest of the team was 1.84 m.

The information about the anthropometric characteristics of the players (body height, weight, and body mass index) and the reach capacities of the spike and block serve as reference values in the selection and training process of the players. When players reach a performance age in volleyball, these values orient the coach in team management. Age also allows one to have a temporal reference of the approximate time until optimal performance may be achieved. Traditionally, the so-called “ten-year rule” (Simon and Chase, 1973) has been used to establish the age of peak performance, and the literature has indicated that the peak performance age in volleyball is 22–26 years of age (Gualdi-Russo and Zaccagni, 2001; Platonov, 1988) (Table 2). The purpose of this paper was to study the ranges of body height, weight, age, spike reach, and block reach of the players in relation to the player position and the level of their respective teams in peak performance volleyball.

## Material and Methods

The analysed sample included 1440 of the 1465 males and 1459 of the 1465 female players who participated in the volleyball competitions of the 2000 Olympic Games, 2002 World Championship, 2004 Olympic Games, 2006 World Championship, 2008 Olympic Games, 2010 World Championship, and 2012 Olympic Games (Table 3). Players’ information was obtained from the databases of the different championships on the official FIVB website (www.fivb.org).

A descriptive, correlational, and longitudinal design was used. The variables studied were as follows: player position (setter, middle blocker, outside-hitter or swing-spiker, opposite, or libero), level of the team (level 1, classified 1st - 4th; level 2, classified 5th - 8th; or level 3, classified 9th to the last position), body height (m), weight (kg), body mass index (weight in kg divided by body height squared in m), spike reach (m), block reach (m), and age of the player (yrs). FIVB classification and criteria for player positions were used.

Descriptive and inferential analyses of the data were done using the software SPSS v.15. A one-way ANOVA was performed to analyse the levels of performance and the player’s role, and when equal variances were found, it was followed up with Scheffe post-hoc testing; however, when unequal variances were found, the Brown-Forsythe Test with Dunnett’s T3 post-hoc testing was performed. To study the differences between genders, the Student t test was used. Statistical significance was set at p<0.05.

## Results

With regard to the evolution of the anthropometric, physical, and age differences between championships (Table 4), in general, a slight increase in body height and a slight decrease in weight, body mass index (BMI), spike reach, and block reach were observed. With regard to gender, significant differences were found for all variables between male and female players (p<0.001). For female players, a significant reduction of the values was found between the 2000 Olympic Games and the 2006 World Championship. Regarding the player position, the results did not show significant variation in the studied variables during the years that were analysed. Significant differences were found for male liberos between the Olympic Games and the World Championship, as the liberos in the last Championship were an average of two years younger (30.1 years vs. 27.9 years) and 0.06 m shorter (1.92m vs. 1.85m). Certain differences between the Olympic Games and the World Championship were found: a) for the spike and block reaches in the libero; and b) for the spike reach in female middle-blockers and outside-hitters, as these players had a 0.06–0.07 m higher reach in the Olympic Games (among 12 classified teams) than in the World Championship (among 24 classified teams). This allows one to combine the data from the players that participated in the competitions to analyse the variables by a player position and by the level of performance; although, the conclusions about the liberos’ body height and age as well as the spike reach of female middle-blockers and outside-hitters have to be analysed carefully.

With regard to body height (Table 5), for both males and females, the tallest players were middle blockers, followed by opposites, outsides, setters, and liberos. There were significant differences between all player positions (for males, p<.001; for females, p<.000), except for opposites and outsides for both genders and opposites and middle blockers for females. For females, no significant differences were found between middle-blockers and opposites. For males, level 1 had a tendency to have the tallest players in all positions, although, these differences were only statistically significant in comparison with players of level 3 (differences of zero to 0.15 m) (p<.02). For females, the teams in level 1 had the tallest players for all positions. These differences were statistically significant between middle-blockers (p<.05), and opposites (p<.005) from levels 1 and 3. For all players positions, females were significantly shorter than males (−0.14m) (p<.001).

With regard to weight (Table 6), both males and females presented similar tendencies. For males, the heaviest players were middle-blockers, followed by opposites, outside-hitters, setters and liberos. Significant differences between all players were found (p<.001), except for middle-blockers and opposites. For females, the heaviest players were middle-blockers, followed by outside-hitters, opposites, and then setters and liberos. There were significant differences between opposites and outside-hitters (p<.010), middle-blockers and outside-hitters (p<.010), as well as setters and liberos (p<.000). By the level of performance, as long as males were concerned, middle blockers of level 2 were significantly heavier than middle blockers of level 3 (1.8 kg) (p<.001). In case of female players, the higher the level of the team was, the higher the weight of the players of all positions (a difference of 2–3 kg was observed). However, these differences were only statistically significant between players in levels 1 and 3 and among outside-hitters and opposites (p<0.027). For all player positions, females were significantly lighter than male players (−18.3 kg) (p<.001).

The values for the BMI showed the same tendencies as body height and weight both by gender and by the level of performance (Table 7). For males, middle-blockers had a statistically significantly lower BMI than the rest of the players: setters (p<0.002), outside hitters (p<0.011), opposites (p<0.000) and liberos (p<0.009). For females, opposites had a statistically significantly lower BMI than the rest of the players: setters (p<0.003), outside hitters (p<0.003), and liberos (p<0.003). Also, significant differences between middle-blockers and liberos were found (p<0.003). By the level of performance, no significant differences were found for male or female players in the different positions. For all player positions, females’ BMIs were significantly lower than males’ BMIs (−1.7 kg/m2) (p<.001).

In relation to the spike reach (Table 8), for both males and females, the players that had the highest reach were middle-blockers and opposites, followed by outside-hitters and then setters and liberos. There were significant differences between all player positions except for middle-blockers and opposites for both males and females. By the level of performance, for males, the players of level 1 and level 2 had higher reaches than players of level 3 (p<.02). However, these differences were only statistically significant for the opposite. For females, when the team’s level was higher, all players had a higher spike reach. The increase by the team’s level was statistically significant between the players of all positions from levels 1 and 2 with the players from level 3 (p<0.05). For all player positions, the females’ spike reach was significantly lower than males’ (−0.41 m) (p<.001).

In relation to the block reach (Table 9), for both males and females, it was observed that players that had the highest block reach were middle-blockers and opposites followed by outside-hitters, and then by setters and liberos. For both males and females, there were significant differences between all player positions except for middle-blockers and opposites, as well as opposites and outside-hitters for males. By the level of performance, no significant differences were found in any position for male players. For females, teams at a higher level had players at all positions with a higher block reach. These differences were significant between the players in levels 1 and 2 with the players of level 3 in all positions (p<0.05) except for the libero position. For all player positions, the females’ block reach was significantly lower than in male players (−0.36 m) (p<.001).

With regard to age (Table 10), for males, liberos and setters were significantly older than middle-blockers and outside-hitters (p<.009). Total values (without regard to the playing position) showed that at higher levels of performance, the players were older. These differences were only significant between levels 1 and 3 (p<.000). For females, no significant differences were found. By the level of performance, the average age of players of level 1 was significantly higher than players of level 3 (p<.001). For males, setters and outside-hitters in level 1 were significantly older than the ones in level 3 (p<.001). For females, middle blockers and liberos in level 1 were significantly older than the ones in level 3 (p<.05). No significant differences were found in the rest of the player positions. For all player positions, the age of the females was significantly lower than that of the male players (−1.7 years old) (p<.001).

## Discussion

The purpose of this study was to provide reference values for body height, weight, age, spike reach, and block reach of volleyball players in relation to the player’s position and the level of their respective teams in peak performance. Data provided give a general image of the players’ characteristics with respect to their position. Role specialisation influences players’ anthropometric and physical characteristics in male and female volleyball players. The values found for body height, weight, and the BMI presented similar tendencies and showed different characteristics according to the player specialisation (Table 1).

For males, two groups of player types can be differentiated. On one side there are middle-blockers, outside-hitters, and opposites, who block and spike. They are taller, heavier, have lower or similar BMIs, have a higher jump reach, and are younger when compared to other players. On the other side there are setters and liberos, who participate less or do not participate in blocking and spiking. They are shorter, lighter, an- Incld older. These results confirm previous studies in relation to the player’s position and physical capacities among males (Gualdi-Russo and Zaccagni, 2001; Marques et al., 2009; Sheppard et al., 2009; Fattahi et al., 2012). Teams work like a system, and every player has a role. The role of each player affects his or her characteristics. Middle-blockers and opposites have a critical role in spiking and blocking. Therefore, their height is critical given that eighty-three percent of players’ reach ability is conditioned by their anthropometric characteristics and their take-off and reach positions (Vint, 1994). This is why these players are the ones that had the highest spike and block reaches. Along these lines, middle-blockers and opposites had higher BMIs due to a higher percentage of muscle, which contributes to their jump reach. These positions also involve more physical implication in the game (e.g. number of jumps), and it may be the reason these players are younger. Of these players, the outside-hitter is the shortest and lightest, probably because this player also has an important role in serve reception, requiring greater court mobility. Setters and liberos, who play in positions where game-organization and reception as well as defence are critical, have different characteristics. On average, they are older players, probably due to their role requiring a higher capacity for reading the game, game analysis, and decision-making. In addition, their body height and jump reach are lower (approx. 0.10 m), because their contribution is lower or null in spiking and blocking.

By the level of performance, for males, the data do not show that at higher levels, the players are taller, lighter, or have lower BMIs. Body height, weight, and the BMI of the different players, by their playing position, are similar and homogeneous at the different levels of classification of the Olympic Games and World Championship teams. This may be because in the Olympics and the World Championship the performance level of teams and players is similar. However, general differences were found in age and a classification position. The higher the level of the team, the older and more experienced their players are. This tendency was also observed for the setter and outside players. Previous studies have shown differences in the physical characteristics of the players regarding their level of performance (Gualdi-Russo and Zaccagni, 2001), but the samples that were compared were from different levels of competition (national first division vs national second division). The sample was composed of national teams that participated in the Olympic Games and World Championship. At the level of competition that was studied, experience is a key factor for the team’s success in men’s volleyball, due to physical characteristics being similar between the teams of different final classification. In addition, these results show the importance of managing and organising the reception and setting in men’s volleyball.

For females, two groups can also be differentiated regarding their physical characteristics, according to whether they have blocking and spiking responsibilities. Middle-blockers, outside-hitters and opposites are taller, heavier, and have higher jump reaches than setters and liberos. These results confirm previous studies in relation to the player position among females (Gualdi-Russo and Zaccagni, 2001; Malousaris et al., 2008; Zhang, 2010; Carbajal et al., 2012; Martin-Matillas et al., 2013). The same aforementioned explanations for males are applicable to females. However, several aspects should be emphasised that are different for female players. Due to the fact that the role of the opposite player is different for female teams (e.g. in some teams they take part in reception), opposites have similar characterises to outside-hitters in women’s volleyball. They are players who are very mobile on the court, who receive and are also at the net to block and spike. However, this aspect can vary in relation to the team that is being analysed (Ejem, 2001; Fröhner, 1997). Data provided in this study are general, due to the main purpose of this study being to provide reference values.

By the level of performance, for females, the best teams have middle-blockers, outside-hitters, and opposites that are taller, heavier, and have better jump reaches. This tendency is different from the one found for male players. For females, the best teams have players with better physical capacities. This could be one of the reasons for different performances by these teams, as the spike and block are the actions that are most highly correlated with winning in volleyball (Palao et al., 2004; Palao et al., 2009). Regarding the average age, similar values were found between different levels of classification. The differences found in middle blockers and liberos between level 1 (1^st^ – 4^th^) and level 3 (9^th^ – last) could show the importance for the teams in level 1 to put players with the most experience in the specialised positions of passing and defence and blocking. These findings confirm previous studies which focused on players’ performance levels (Gualdi-Russo and Zaccagni, 2001): the higher the level of the team, the higher physical capacities of their players. The data show that the level of the teams that participate in the Olympic Games and World Championship is more diverse in the women’s game than in the men’s game. At this level of competition, physical characteristics have key importance with regard to the team’s success in women’s volleyball.

This study provides reference values to guide the players’ selection, understand game dynamics, and understand the role of the different players in men’s and women’s volleyball. Players’ characteristics are a result of the selection process (natural and intentional) and specific training done in practice and competition by the players. The information about the players that have reached this level of competition can be used as criteria in the multifactorial process of talent selection in volleyball. Examples of the application of these data could be the establishment of levels to achieve in physical tests such as the spike reach. The data also provide information about the importance of the different aspects of the game or the reasons for their importance. At this level, the data found show higher average values in the spike reach than in the block reach (0.20–0.30 m for males and 0.15–0.25 m for females). These differences could be one of the reasons for the superiority of the spike over the block in volleyball (Eom and Schutz, 1992; Palao et al, 2004; 2007), and they are probably caused by previous displacement of the players in the approach run and different positions in the reach (in spiking the reach is with one hand, and in blocking it is done with two hands) (Vint, 1994).

The data show different tendencies in men’s and women’s volleyball regarding physical capacities and age, as well as the range of age in which players obtain the best results. At this level, men who obtained their best result were older than women by 2–3 years. No reason can be given about the differences between men and women in this regard, though it may show an early start or early specialisation in the sport. From a general perspective, the results indirectly show that a player’s best performance is approximately 8–12 years after the theoretical specialisation age (16 years old or later) and even later for setters and liberos. The so-called “ten-year rule” is not followed when it is taken as a reference from the theoretical starting age in volleyball (9–12 years old). These results seem to indicate that long specialised training after general training is necessary. The acquisition of training and competitive experience during a minimum of 10–12 years is necessary to achieve peak performance, in addition to the ideal anthropometrics and physical capabilities.

## Conclusions

The results show differences between body height, spike, and block reaches, and the age of the players by their position. These differences are related to the needs of the different positions with regard to the actions they perform. Middle-blockers, opposites, and outside-hitters have characteristics that are more conducive to blocking and spiking (taller, lower BMIs, higher reach, and younger), and setters and liberos have characteristics that may be more suitable for setting, receiving, and digging (smaller, lighter, and older). Player’s age was a variable that differentiated first teams at this level of competition for males, and physical capacities (height, weight, spike reach, and block reach) were variables that differentiated first teams at this level of competition for females.

The results give reference values of the anthropometric characteristics, physical capacities, and age of the peak performance player to orientate the selection and training process. Additionally, the data provide information that can help to understand game dynamics and the role of the different players in men’s and women’s volleyball. Future studies should evaluate the type of technique by a player position (occurrence and efficacy) in order to give reference values to be utilised in practice.

## Figures and Tables

**Figure 2:**
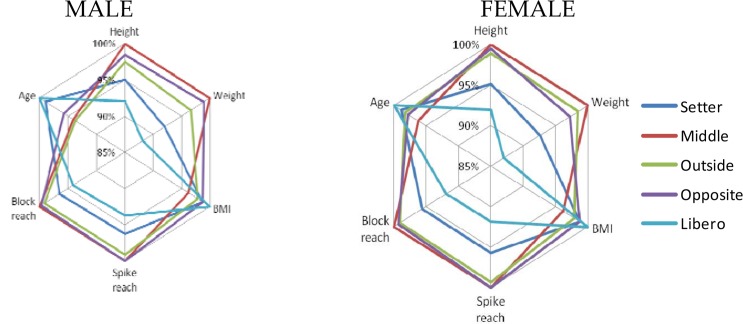
Players’ characteristics regarding their role.

**Figure 3:**
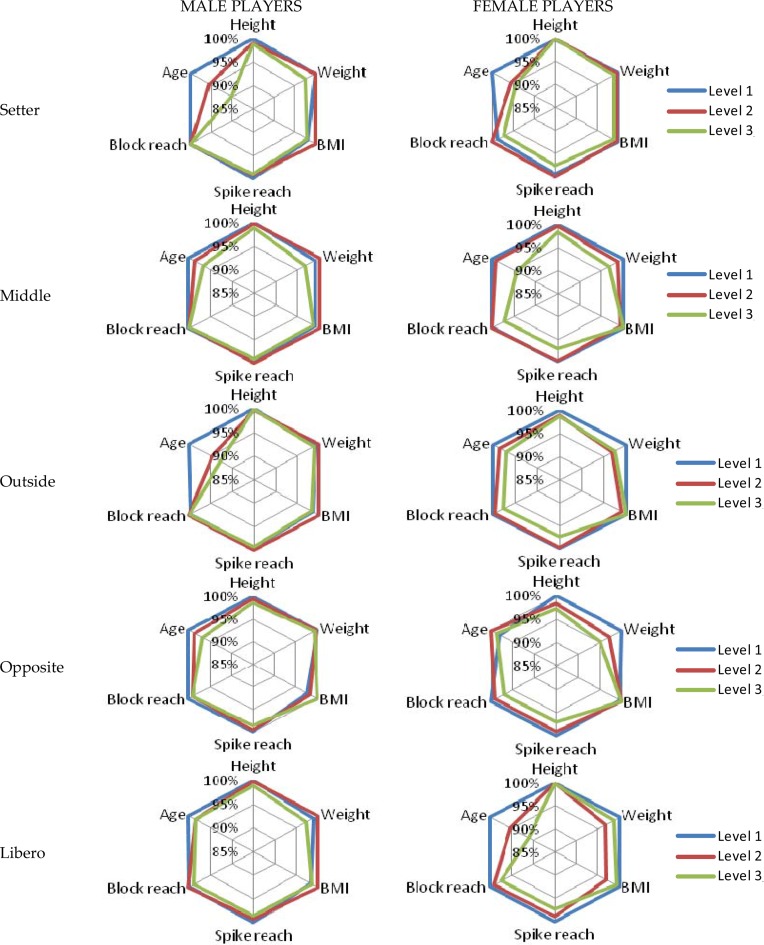
Players’ characteristics regarding their role and team’s classification.

**Table 1:** Functions and responsibilities of the players regarding their position

Position	Actions	Action’s intensity, respectively
Setter	Setting, blocking, and defence	sub-max, max, and sub-max
Middle blocker	Spiking, blocking, and defense^1^	max, max, and sub-max
Outside	Reception, spiking, blocking, and defence	sub-max, max, max, and sub-max
Opposite	Spiking, blocking, and defense^1^	max, max, and sub-max
Libero	Reception and defence	sub-max and sub-max

1 In women’s volleyball, the middle blocker and the opposite can also play a role in reception.

**Table 2:** Body height, weight, and age differences by a player position in Italian, Greek, Chinese, Cuban and Spanish volleyball teams

		Italian ^1^		Greek ^2^	Chinese ^3^	Cuban ^4^	Spanish^5^
Position		Male	Female	Female	Female	Female	Female
n		234	244	163	100	41	147
Setter	Body height	1.85	1.72	1.77	1.81	1.78	1.76
	Weight	81.0	67.8	67.8	68.5	73.7	66.7
	Age	24.8	23.1		22.1	23.6	
Middle-blocker	Body height	1.96	1.78	1.82	1.88	1.87	1.84
	Weight	91.0	71.0	74.3	70.3	79	74.8
	Age	25.1	23.6		21.9	21.8	
Outside	Body height	1.91	1.78	181.2	1.85	1.80	1.80
	Weight	87.1	72.5	72.8	75.6	74.5	72.6
	Age	24.4	22.9		23	23.2	
Opposite	Body height	1.94	1.79	1.84	1.84		1.83
	Weight	91.5	72.6	71.4	68.2		78.3
	Age	24.3	22.3		22.6		
Líbero	Body height			1.71	1.75		1.69
	Weight			63.3	66.2		65.6
	Age				21.5		

Average	Body Height	1.93	1.78	1.80	1.84	1.82	1.80
	Weight	88.4	71.2	71.0	70.5	75.2	72.3
	Age	24.5	23.4	25.7	22.3	23.1	24.8

Body height is given in metres, weight in kilograms, and age in years.

1 Data are from players who participated in the Italian League (first and second divisions) in the 1992–93 and 1993–94 seasons. (Gualdi-Russo and Zaccagni, 2001).

2 Data are from players who participated in the Greek League (first and second divisions) in the 2002–03 season (Malousaris et al,. 2008).

3 Data are from players from the top eight teams who participated in the Chinese National League in 2007–08 (Zhang, 2010).

4 Data are from members of the Cuban national team that participated in the Summer Olympic Games in Barcelona 1992, Atlanta 1996, and Sydney 2000 (Carvajal et al., 2012).

5 Data are from players who participated in the Spanish League (first division) in the 2003–04 season (Martin-Matillas et al., 2013).

**Table 3:** Number of teams and players positions analyzed by competitions of the Olympic Games and World Championships between 2000 and 2012

Gender	Competition	Teams	Setter	Middle	Outside	Opposite	Libero	Total
Male	2000 OG	12	24	37	41	24	14	140
	2002 W Ch	24	48	71	105	44	19	287
	2004 OG	12	24	37	45	26	12	144
	2006 W Ch	24	45	79	107	33	23	287
	2008 OG	12	22	37	52	21	12	144
	2010 W Ch	24	48	50	85	77	34	294
	2012 OG	12	24	36	48	23	13	144

	Total men	120	235	347	483	248	127	1440

Female	2000 OG	12	22	38	50	15	12	137
	2002 W Ch	24	45	80	103	32	28	288
	2004 OG	12	24	42	48	19	11	144
	2006 W Ch	24	46	79	94	38	31	288
	2008 OG	12	27	46	44	15	12	144
	2010 W Ch	24	49	37	96	88	44	314
	2012 OG	12	24	38	47	18	17	144

	Total women	120	237	360	482	225	155	1459

	Totals 240		472	707	965	473	282	2899

*Data are from players who participated in the competitions of the 2000 Olympic Games, 2002 World Championship, 2004 Olympic Games, 2006 World Championship, 2008 Olympic Games, 2010 World Championship, and 2012 Olympic Games*.

**Table 4:** Body height, weight, body mass index (BMI), spike reach, block reach, and age of volleyball players who participated in the Olympic Games and World Championships between 2000 and 2012

Gender	Competition	Height	Weight	BMI	Spike reach	Block reach	Age
Male^*^	2000 OG	1.97±0.06	90.0±7.7	23.2±1.6	3.44±0.11	3.26±0.11	26.4±3.6
	2002 W Ch	1.96±0.06	88.3±7.5	22.9±1.6	3.43±0.12	3.26±0.12	26.8±4
	2004 OG	1.97±0.07	89.4±7.2	23.0±1.4	3.43±0.13	3.24±0.11	28.2±4**^+^**
	2006 W Ch	1.97±0.07	88.5±8.1	22.8±1.5	3.42±0.14	3.26±0.12	26.8±3.9**^+^**
	2008 OG	1.97±0.07	88.9±7.8	22.8±1.5	3.45±0.12	3.27±0.12	28.1±4.4**^+^**
	2010 W Ch	1.96±0.08	87.7±8.7	22.8±1.8	3.41±0.14	3.24±0.13	28.5±4.3**^+^**
	2012 OG	1.98±0.07	89.7±8.6	23.0±1.8	3.43±0.13	3.23±0.13	27.0±4.6**^+^**

	Average	1.97±0.07	88.7±8.1	22.9±1.6	3.43±13	3.25±0.12*	27.4±4.2*

Female^*^	2000 OG	1.82±0.07	71.7±6.6	21.8±1.7**^+^**	3.05±0.12**^+^**	2.90±0.13	25.3±4.1
	2002 W Ch	1.82±0.07	70.4±7.0	21.3±1.8	3.01±0.12**^+^**	2.88±0.14	24.8±4.0**^+^**
	2004 OG	1.82±0.08	71.6±6.5	21.5±1.8	3.03±0.14	2.91±0.15	26.1±4.1**^+^**
	2006 W Ch	1.82±0.08	69.9±6.6	21.1±1.7**^+^**	3.00±0.14**^+^**	2.88±0.15	24.8±4.2**^+^**
	2008 OG	1.83±0.08	70.1±6.7	20.9±1.6**^+^**	3.03±0.15	2.91±0.15	25.6±4.5
	2010 W Ch	1.83±0.08	69.8±7.2	20.9±1.7**^+^**	3.00±0.15**^+^**	2.88±0.15	27.1±3.8**^+^**
	2012 OG	1.82±0.09	69.5±7.6	20.9±1.7**^+^**	3.01±0.17	2.88±0.17	26.2±4.2**^+^**

	Average	1.82±0.08	70.3±6.9*	21.2± 1.7*	3.01±14.4*	2.89±14.8	25.7±4.2*

Body height is given in meters, weight in kilograms, BMI in kg/m^2^, spike reach in meters, block reach in meters, and age in years.

* Significant differences between gender.

* Significant differences in this variable between positions.

+ Significant differences in this variable between team’s classification.

**Table 5:** Average body height (m) of volleyball players with regard to their position and their team’s level

Gender	Classification	Setter	Middle	Outside	Opposite	Libero	Average
Male^*^	1^st^ – 4^th^	1.93±0.05	2.03±0.04**^+^**	1.98±0.05	2.00±0.06**^+^**	1.87±0.04	1.98±0.07**^+^**
	5^th^ – 8^th^	1.92±0.07	2.03±0.05**^+^**	1.97±0.05	1.99±0.06**^+^**	1.87±0.06	1.97±0.07
	9^th^ – last	1.91±0.06	2.01±0.05**^+^**	1.97±0.05	1.97±0.05**^+^**	1.86±0.06	1.96±0.07**^+^**

	Average	1.92±0.06±	2.02±0.05*	1.97±0.05	1.99±0.06*	1.860.06±	1.97±0.07*

Female^*^	1^st^ – 4^th^	1.77±0.07	1.88±0.04**^+^**	1.86±0.6**^+^**	1.89±0.07**^+^**	1.72±0.07	1.84±0.08**^+^**
	5^th^ – 8^th^	1.77±0.06	1.87±0.04**^+^**	1.84±0.06**^+^**	1.86±0.07	1.71±0.06	1.83±0.08**^+^**
	9^th^ – last	1.78±0.06	1.85±0.06**^+^**	1.83±0.06**^+^**	1.83±0.06**^+^**	1.71±0.07	1.81±0.08**^+^**

	Average	1.77±0.06*	1.86±0.06*	1.84±0.06*	1.85±0.07*	1.71±0.07*	1.82±0.08*

Data are from players who participated in the competitions of the Olympic Games and World Championships from 2000 to 2012.

* Significant differences between gender.

* Significant differences in this variable between positions.

+ Significant differences in this variable between team’s classification.

**Table 6:** Average weight (kg) of volleyball players with regard to their position and their team’s level

Gender	Classification	Setter	Middle	Outside	Opposite	Libero	Average
Male^*^	1^st^ – 4^th^	85.9±6.4	92.9±6.4	89.16.3±	91.4±7.8	81.7±5.5	89.2±7.4
	5^th^ – 8^th^	85.9±6.7	93.9±7.9**^+^**	89.7±7.1	91.2±7.2	82.6±6.7	89.8±7.9**^+^**
	9^th^ – last	83.9±7.6	90.9±8.1**^+^**	88.7±7.2	90.9±8.1	80.3±7.0	88.0±8.3**^+^**

	Average	84.8±7.1*	92.1±7.8*	89.0±7.0*	91.1±7.8*	81.2±6.6*	88.7±8.0*

Female^*^	1^st^ – 4^th^	67.9±6.0	74.4±6.0**^+^**	73.4±6.7**^+^**	73.3±5.8**^+^**	64.4±8.1	71.8±7.17**^+^**
	5^th^ – 8^th^	67.8±5.2	73.4±5.0	71.0±5.8**^+^**	71.3±6.0	62.3±6.4	70.3±6.38**^+^**
	9^th^ – last	67.2±5.4	72.0±7.4**^+^**	71.5±6.3**^+^**	69.7±6.5**^+^**	63.5±5.7	69.7±7.0**^+^**

	Average	67.5±5.5*	72.9±6.6*	71.8±6.3*	70.9±6.4*	63.46.4±	70.3±6.9*

Data are from players who participated in the competitions of the Olympic Games and World Championships from 2000 to 2012.

* Significant differences between gender.

* Significant differences in this variable between positions.

+ Significant differences in this variable between team’s classification.

**Table 7:** Average body mass index (kg/m)^2^ of international volleyball players with regard to their position and their team’s level

Gender	Classification	Setter	Middle	Outside	Opposite	Libero	Average
Male^*^	1^st^ – 4^th^	23.0±1.5	22.5±1.6	22.8±1.4	22.8±1.7	23.3±1.1	22.8±1.5**^+^**
	5^th^ – 8^th^	23.4±1.3	22.8±1.7	23.1±1.5	23.0±1.5	23.6±1.5	23.1±1.5**^+^**
	9^th^ – last	23.0±1.7	22.5±1.7	22.8±1.7	23.3±1.7	23.3±1.4	22.9±1.7

	Average	23.1±1.6*	22.5±1.7*	22.9±1.6*	23.1±1.7	23.4±1.4	22.9±1.6*

Female^*^	1^st^ – 4^th^	21.6±1.4	21.0±1.7	21.3±1.7	20.7±1.6	21.8±2.0	21.2±1.7
	5^th^ – 8^th^	21.6±1.5	20.1±1.4	21.1±1.5	20.7±1.5	21.2±1.4	21.1±1.5
	9^th^ – last	21.4±2.0	21.0±2.0	21.3±1.7	20.7±1.8	21.7±1.7	21.2±1.9

	Average	21.5±1.8*	21.0±1.8*	21.3±1.6*	20.7±1.7*	21.6±1.7*	21.2±1.7

Data are from players who participated in the competitions of the Olympic Games and World Championships from 2000 to 2012.

* Significant differences between gender.

* Significant differences in this variable between positions.

+ Significant differences in this variable between team’s classification.

**Table 8:** Average spike reach (m) of international volleyball players with regard to their position and their team’s level

Gender	Classification	Setter	Middle	Outside	Opposite	Libero	Average
Male^*^	1^st^ – 4^th^	3.37±0.10	3.49±0.14	3.45±0.10	3.50±0.08**^+^**	3.29±0.12	3.44±0.13**^+^**
	5^th^ – 8^th^	3.34±0.12	3.50±0.10	3.47±0.09	3.49±0.11	3.27±0.12	3.44±0.13**^+^**
	9^th^ – last	3.34±0.11	3.47±0.13	3.44±0.10	3.45±0.09**^+^**	3.24±0.14	3.41±0.13**^+^**

	Average	3.35±0.11*	3.48±0.12*	3.45±0.10*	3.48±0.10*	3.26±0.13*	3.43±0.13*

Female^*^	1^st^ – 4^th^	2.96±0.13**^+^**	3.12±0.09**^+^**	3.09±0.09**^+^**	3.12±0.09**^+^**	3.88±0.15**^+^**	3.06±0.13**^+^**
	5^th^ – 8^th^	2.98±0.11**^+^**	3.12±0.08**^+^**	3.09±0.11**^+^**	3.08±0.11**^+^**	3.85±0.18	3.05±.014**^+^**
	9^th^ – last	2.90±012**^+^**	3.03±0.11**^+^**	3.01±0.11**^+^**	3.02±0.12**^+^**	2.80±0.15**^+^**	2.98±0.14**^+^**

	Average	2.94±0.13*	3.07±011*	3.05±0.11*	3.07±0.12*	2.82±0.16*	3.01±0.14*

Data are from players who participated in the competitions of the Olympic Games and World Championships from 2000 to 2012.

* Significant differences between gender.

* Significant differences were found in this variable between positions.

+ Significant differences in this variable between team’s classification.

**Table 9:** Average block reach (m) of international volleyball players with regard to their position and their team’s level

Gender	Classification	Setter	Middle	Outside	Opposite	Libero	Average
Male^*^	1^st^ – 4^th^	3.18±0.09	3.30±0.08	3.26±0.11	3.32±0.09	3.12±0.11	3.25±0.11
	5^th^ – 8^th^	3.18±0.11	3.30±0.09	3.27±0.10	3.28±0.28	3.12±0.09	3.25±0.12
	9^th^ – last	3.18±0.11	3.30±0.11	3.27±0.10	3.29±0.11	3.09±0.12	3.25±0.12

	Average	3.18±0.11*	3.30±0.10*	3.27±0.10*	3.29±0.11*	3.10±0.11*	3.25±0.12

Female^*^	1^st^ – 4^th^	2.84±0.15**^+^**	2.99±0.11**^+^**	2.96±0.11**^+^**	2.98±0.10**^+^**	2.76±0.16	2.93±0.14**^+^**
	5^th^ – 8^th^	2.87±0.14**^+^**	2.99±0.11**^+^**	2.95±0.13**^+^**	2.96±0.12**^+^**	2.74±0.18	2.93±0.15**^+^**
	9^th^ – last	2.79±0.13**^+^**	2.91±0.11**^+^**	2.89±0.12**^+^**	2.90±0.13**^+^**	2.69±0.14	2.86±0.14**^+^**

	Average	2.82±0.14*	2.95±0.11*	2.92±0.12*	2.93±0.13*	2.71±0.16*	2.89±0.15*

Data are from players who participated in the competitions of the Olympic Games and World Championships from 2000 to 2012.

* Significant differences between gender.

* Significant differences were found in this variable between positions.

+ Significant differences in this variable between team’s classification.

**Table 10:** Average age (yrs) of volleyball players with regard to their position and their team’s level

Gender	Classification	Setter	Middle	Outside	Opposite	Libero	Average
Male^*^	1^st^ – 4^th^	30.3±4,7**^+^**	27.5±3.8	28.4±4.0**^+^**	28.0±4.1	29.0±3.6	28.5±4.1**^+^**
	5^th^ – 8^th^	29.0±4.8	27.2±3.7	26.9±3.6**^+^**	27.6±4.6	28.6±4.1	27.6±4.1**^+^**
	9^th^ – last	27.4±3.9**^+^**	26.6±4.1	26.3±4.1**^+^**	27.1±4.0	28.6±4.2	26.9±4.1**^+^**

	Average	28.4±4.5*	27.0±3.9*	26.9±4.1*	27.5±4.2	28.7±4.0*	27.4±4.2*

Female^*^	1^st^ – 4^th^	27.0±4.4	26.0±4.0**^+^**	26.2±3.4	25.4±3.1	28.0±4.0**^+^**	26.3±3.8**^+^**
	5^th^ – 8^th^	25.8±3.9	25.7±4.1	25.9±4.1	25.9±3.6	26.6±4.7	25.9±4.1
	9^th^ – last	25.4±4.3	24.6±4.0**^+^**	25.5±4.5	25.6±4.4	25.5±4.0**^+^**	25.3±4.3**^+^**

	Average	25.9±4.2	25.2±4.1*	25.7±4.2	25.6±3.9	26.2±4.2*	25.7±4.2*

Data are from players who participated in the competitions of the Olympic Games and World Championships from 2000 to 2012.

* Significant differences between gender.

* Significant differences were found in this variable between positions.

+ Significant differences in this variable between team’s classification.
